# Factors associated with handwashing behaviour among rural schoolchildren: a cross-sectional study in Georgia, 2024

**DOI:** 10.7717/peerj.21502

**Published:** 2026-07-14

**Authors:** Mariam Izoria, Natia Kakutia, Levan Metreveli, Levan Baramidze, Luiza Gabunia, Nino Gvajaia

**Affiliations:** 1 Department of Public Health, Tbilisi State Medical University, Tbilisi, Georgia; 2 Department of Epidemiology and Biostatistics, Tbilisi State Medical University, Tbilisi, Georgia; 3 Department of Global Health, University of Georgia, Tbilisi, Georgia; 4 Department of Health Policy, Management and Economics, Tbilisi State Medical University, Tbilisi, Georgia; 5 Department of Pharmacology, Tbilisi State Medical University, Tbilisi, Georgia; 6 Department of Medicine, Tbilisi State Medical University, Tbilisi, Georgia

**Keywords:** Handwashing, Hand hygiene, Schoolchildren, Rural schools, WASH, Cross-sectional study, Behaviour change, COM-B model, Georgia

## Abstract

**Background:**

Handwashing is a critical measure for reducing diarrhoeal and respiratory infections among school-aged children, yet maintaining consistent practice remains a challenge. Behavioural factors, including daily routines, environmental cues, and perceptions of hygiene spaces, shape handwashing behaviour beyond the availability of water and soap. This study examined the behavioural determinants of handwashing among rural schoolchildren in Georgia using the Capability, Opportunity, Motivation-Behaviour (COM-B) model.

**Methods:**

A cross-sectional survey was conducted in 2024 among 368 students from 10 rural public schools in the Adjara region. A structured interviewer-administered questionnaire assessed sociodemographic characteristics, knowledge, environmental conditions, motivational factors, and self-reported handwashing practices. Adequate handwashing was defined as washing hands both before eating and after toilet use. Variables associated with adequate behaviour in bivariate analyses (*p* < 0.20) were considered for inclusion in a multivariable logistic regression model.

**Results:**

Overall, 85.9% of students reported adequate handwashing behaviour. Although knowledge of correct handwashing steps was high (91.3%), substantial behavioural gaps were observed. In the adjusted model, three factors independently predicted adequate handwashing: not relying solely on washing when hands were visibly dirty—indicating a stable hygiene routine (AOR = 30.3, 95% CI [13.0–71.4]); perceiving the school toilet environment as comfortable or safe (AOR = 6.6, 95% CI [1.8–23.8]); and washing hands at least five times per day (AOR = 2.22, 95% CI [1.04–4.74]). Water, adequate Sanitation, and proper Hygiene (WASH) education showed a borderline association (AOR = 2.15, *p* = 0.060). Knowledge-related indicators and availability of water or soap were not significant predictors in the multivariable analysis.

**Conclusion:**

Handwashing behaviour among rural schoolchildren in Georgia is driven less by knowledge or basic facility availability and more by routine formation, perceived comfort of hygiene spaces, and motivational factors. Strengthening handwashing habits, improving the qualitative aspects of school toilets, and incorporating behaviourally informed approaches into hygiene education may enhance the sustainability of WASH outcomes. Applying the COM-B model provides guidance for designing interventions that support consistent, protective hygiene practices in school settings.

## Introduction

Handwashing is one of the most effective public health measures for preventing infectious diseases among school-aged children, yet ensuring consistent practice remains a challenge in many settings. Children’s handwashing does not depend solely on the availability of soap and water; it is also shaped by daily experiences, behavioural cues, and social expectations within the school environment (Contzen & Mosler, 2015; White et al., 2020; Dreibelbis et al., 2016). Even when facilities are adequate, students may not wash their hands regularly unless hygiene is socially expected, prompted by routine cues, or reinforced by teachers and peers.

Globally, inadequate hand hygiene contributes substantially to the burden of diarrhoeal and respiratory infections among children. Handwashing with soap can reduce the incidence of diarrhoea by 30–48% (Ejemot-Nwadiaro et al., 2021; Freeman et al., 2014) and is also associated with reduced influenza-like illness and acute respiratory infections in school settings (Rabie & Curtis, 2006). Because schools are high-contact environments where pathogens spread quickly, insufficient handwashing can have immediate consequences for children’s health, absenteeism, and wellbeing.

In Georgia, efforts to improve school Water, adequate Sanitation, and proper Hygiene (WASH) infrastructure have accelerated in recent years. National reviews by the Ministry of Education, UNICEF Georgia (2022), and the National Center for Disease Control and Public Health (NCDC) (2021) have documented recurring gaps in the reliability of water supply, the cleanliness of school toilets, and the integration of hygiene education into the curriculum, particularly in rural regions. However, these assessments have largely focused on infrastructure, with limited attention to the behavioural determinants that influence whether students consistently practise handwashing during the school day. Given that schools are high-contact environments where respiratory and gastrointestinal infections can spread rapidly, understanding these behavioural factors is essential for effective prevention. Behavioural science emphasises the need to address knowledge, environmental conditions, and motivation together. Interventions that combine education with behavioural strategies, such as prompts near sinks, structured routines, and peer modelling, have demonstrated greater improvements in handwashing than infrastructure-only approaches (World Health Organization (WHO), 2009; UNICEF, 2024; Watson et al., 2021; Garn et al., 2013). This highlights the need to examine not only whether students are able to wash their hands, but also whether they feel motivated and supported to do so consistently within their school context.

The Capability, Opportunity, Motivation-Behaviour (COM-B) model provides a practical framework for understanding these influences. According to the COM-B model, behaviours such as handwashing occur when individuals have the capability (knowledge and skills), the opportunity (a supportive physical and social environment), and the motivation (beliefs, habits, and intentions) required to perform the behaviour regularly (Michie, Van Stralen & West, 2011). The COM-B model has increasingly been applied in WASH and hygiene behaviour research to better understand how environmental conditions, behavioural routines, and social influences interact to shape handwashing practices (Contzen & Mosler, 2015; Dreibelbis et al., 2016; White et al., 2020). However, most studies applying behavioural frameworks to handwashing have been conducted in low-income settings in Africa or Asia, whereas evidence from Eastern Europe and the Caucasus region remains limited. In Georgia, research on school WASH has primarily focused on infrastructure and facility availability rather than on behavioural determinants. Applying the COM-B framework in this context therefore provides an opportunity to examine how capability, opportunity, and motivation interact to influence students’ handwashing behaviour in rural school settings.

This study investigates the behavioural determinants of handwashing among rural schoolchildren in Georgia. Although previous national assessments have primarily focused on the availability of WASH infrastructure, limited evidence exists on the behavioural drivers that influence whether students actually wash their hands at critical moments during the school day. In particular, few studies in the Caucasus region have applied behavioural theory to examine how capability, opportunity, and motivation interact to shape hygiene behaviour in school settings. By applying the COM-B framework to rural schools in Georgia, this study aims to address this gap and provide behaviourally informed evidence to support more effective school-based hygiene promotion.

## Materials & Methods

### Study design, setting, and participants

This cross-sectional study used data from a broader school WASH assessment conducted in rural communities of the Adjara region in 2024. Ten public schools were selected in collaboration with the Public Health Center of the Autonomous Republic of Adjara to represent rural municipalities within the region. Selection was based on geographic coverage and willingness to participate; therefore, a convenience sampling approach was applied at the school level.

Within each participating school, all students present on the day of data collection who had parental consent and provided assent were invited to participate in the survey. No additional exclusion criteria were applied. The survey was administered primarily to students in grades VII–XII, as the study aimed to explore the behavioural determinants of handwashing among students who spend longer periods independently in school environments. A small number of students from grades I–VI were also included when present and eligible; therefore, younger primary school students were underrepresented in the sample.

The final analytical sample included 368 students who completed the questionnaire. As participation depended on school attendance and consent procedures, the sample represents students who were available at the time of the survey rather than a randomly selected population sample. Because the survey was designed as an exploratory behavioural assessment of school WASH conditions, a formal sample size calculation was not performed prior to data collection.

### Data collection tool and procedures

A structured, interviewer-administered questionnaire was used to collect information on sociodemographic characteristics, handwashing routines, access to hygiene facilities, perceived barriers, exposure to hygiene information, and motivational factors.

Data were collected between March and April 2024 by trained field researchers. Prior to data collection, researchers received standardized training on questionnaire administration, neutral prompting, and maintaining a consistent interview approach across schools. They were instructed to avoid leading explanations, maintain the confidentiality of responses, and ensure that students completed the questionnaire independently.

Questionnaires were administered in small groups in designated classrooms. Interviewers read each item aloud, provided clarification when needed, and ensured that students completed their responses individually. The completion time was approximately 10–15 min per student. Questionnaires were checked for completeness on-site, transported to the study centre, and entered into a secure electronic database, with routine quality checks performed to ensure accuracy.

### Questionnaire development and validity

The questionnaire was developed based on previously published school hygiene and WASH survey instruments, with several items adapted from established school-based WASH studies and international monitoring tools (Freeman et al., 2014; Garn et al., 2013; World Health Organization (WHO), 2009). To align with the Capability, Opportunity, Motivation-Behaviour (COM-B) framework, survey items were conceptually grouped into three behavioural domains: capability (knowledge and understanding of handwashing practices), opportunity (availability and perceived conditions of hygiene facilities), and motivation (habitual behaviour patterns and exposure to hygiene-related messaging). This classification was applied as an analytical framework rather than for the construction of formal psychometric scales.

Prior to the main survey, the questionnaire was reviewed by public health researchers with experience in WASH studies and was piloted among a small group of students to assess clarity, comprehension, and contextual relevance. Minor wording adjustments were made following the pilot test.

Because the questionnaire primarily included factual and behavioural indicators rather than multi-item latent constructs, formal psychometric scale validation, such as internal consistency testing, was not applied.

### Study variables

### Outcome

The primary outcome was adequate handwashing behaviour, defined as self-reported handwashing both before eating and after toilet use, consistent with recommended critical moments for hand hygiene in school settings (Garn et al., 2013; Freeman et al., 2014; WHO & UNICEF, 2015). These two moments are widely used as core behavioural indicators in school-based WASH research because they represent key opportunities for interrupting faecal-oral transmission.

### Explanatory variables

Independent variables were selected *a priori* and organised using the Capability, Opportunity, Motivation-Behaviour (COM-B) framework. Sociodemographic characteristics (age, sex, and school grade) were included as potential confounders. Capability-related measures captured students’ knowledge of correct handwashing practices and their perceived understanding of the importance of handwashing. Opportunity-related variables reflected the school context, including the reported availability of water and soap, perceived cleanliness of toilets, perceived comfort and safety of toilet spaces, and infrastructural barriers (*e.g.*, broken sinks). Motivation-related variables assessed exposure to WASH education, the perceived influence of hygiene messaging, and participation in school-based WASH activities.

### Statistical analysis

Data were analysed using IBM SPSS Statistics (version 16.0; Chicago, IL, USA). Participant characteristics were summarised using frequencies and percentages for categorical variables. Associations between explanatory variables and adequate handwashing were evaluated using chi-square tests. Variables with *p* < 0.20 in the bivariate analyses were considered for entry into a multivariable logistic regression model to identify independent predictors of adequate handwashing behaviour. Results are presented as adjusted odds ratios (AORs) with 95% confidence intervals (CIs). Statistical significance was set at *p* < 0.05.

Participants with missing outcome data were excluded from the outcome analysis. For the multivariable models, listwise deletion was applied because the proportion of missing data among the explanatory variables was small.

Additional diagnostic checks were conducted to assess the stability of the regression estimates. Multicollinearity among explanatory variables was examined using variance inflation factors (VIFs), and no evidence of problematic collinearity was observed (all VIF values < 2). Because students were recruited from ten schools, the potential influence of school-level clustering was also assessed in sensitivity analyses. Including school as a clustering variable did not materially change the direction or statistical significance of the main associations; therefore, the results from the standard logistic regression model are presented.

### Ethical considerations

Ethical approval was granted by the Tbilisi State Medical University Biomedical Research Ethics Committee (106th session; Ref: N5-2023/106; approved on 1 December 2023). Permission for school-based data collection was provided by the Legal Entity of Public Law “Public Health Center of the Autonomous Republic of Adjara” (Letter No. 01-14/03, dated 27 January 2022). Written informed consent was obtained from all adult participants. For participants under the age of 18, written consent was provided by a parent or legal guardian, along with assent from the minor. The consent forms used in this study are available as a confidential Supplementary File.

## Results

A total of 368 students from ten rural public schools participated in the survey. Most respondents were in grades 7–9 (64.1%), and 59.5% were female (Table 1). Knowledge-related indicators were generally high, with most students reporting knowledge of correct handwashing practices and recognising the importance of hand hygiene for disease prevention.

**Table 1:** Sociodemographic and capability-related characteristics of students (*N* = 368).

Variable	Category	*n* (%)
Sex	Female	219 (59.5)
	Male	149 (40.5)
Grade level	1–6	12 (3.3)
	7–9	236 (64.1)
	10–12	120 (32.6)
Knowledge about handwashing	Yes	336 (91.3)
	No	32 (8.7)
Perceived importance of handwashing	Important	364 (98.9)
	Not important	4 (1.1)
Understands that handwashing prevents diarrhoea/infection	Yes	356 (96.7)
	No	12 (3.3)

School hygiene conditions showed greater variability (Table 2).Running water was reported as usually available at school, whereas soap availability was lower. Several infrastructural barriers were also reported, including the occasional lack of water, broken sinks, and unpleasant odours in the toilets. Despite these challenges, most students perceived the school toilets as comfortable or safe.

**Table 2:** Opportunity, motivational, and behavioural characteristics (*N* = 368).

Variable	Category	*n* (%)
Water availability at school	Yes	330 (89.7)
Soap availability at school	Yes	244 (66.3)
Able to wash hands when needed	Yes	277 (75.3)
Reported barriers	Lack of soap	86 (23.4)
	Lack of water	67 (18.2)
	Broken sink	55 (14.9)
	Unpleasant smell	68 (18.5)
Toilet cleanliness	Clean	234 (63.6)
Toilet comfort/safety	Comfortable/safe	349 (94.8)
Exposure to hygiene information	Yes	284 (77.2)
WASH education received	Yes	227 (61.7)
Washes hands when visibly dirty	Yes	83 (22.6)
Daily handwashing frequency	≥5 times/day	239 (64.9)
	1–4 times/day	129 (35.1)
Adequate handwashing behaviour^*^	Yes	316 (85.9)

**Notes.**

* Adequate handwashing behaviour was defined as self-reported handwashing both before eating and after toilet use.

Motivation-related indicators were moderate to high. Most students reported receiving hygiene information at school and washing their hands frequently during the day. Overall, 85.9% of students reported adequate handwashing behaviour, defined as washing their hands both before eating and after toilet use.

In the bivariate analyses (Table 3), adequate handwashing was associated with several capability-, opportunity-, and motivation-related factors. Among the capability indicators, knowledge of correct handwashing practices and understanding the role of handwashing in infection prevention were significantly associated with adequate behaviour. Perceived comfort and safety of toilet facilities showed a strong association among opportunity-related factors. Motivation-related variables, including exposure to WASH education and higher daily handwashing frequency, were also associated with adequate handwashing. In contrast, basic infrastructure indicators such as water availability, soap availability, and toilet cleanliness were not significantly associated.

**Table 3:** Bivariate associations between independent variables and adequate handwashing behaviour (*N* = 368).

Domain	Variable	Adequate handwashing (%)	*p*-value
Capability	Knows correct handwashing steps	87.2 *vs* 71.9	0.017
	Understands handwashing prevents infection	87.6 *vs* 33.3	<0.001
Opportunity	Water availability	87.0 *vs* 76.3	0.074
	Soap availability	88.1 *vs* 81.5	0.083
	Ability to wash hands when needed	86.3 *vs* 84.6	0.692
	Broken sink reported	94.5 *vs* 84.3	0.045
	Toilet comfort/safety	87.7 *vs* 52.6	<0.001
	Toilet cleanliness	86.8 *vs* 84.3	0.521
	Unpleasant smell in toilets	86.8 *vs* 85.7	0.814
Motivation	WASH education received	89.0 *vs* 80.9	0.029
	Daily handwashing frequency (≥5/day)	90.0 *vs* 78.3	0.002
	Washing only when visibly dirty	50.6 *vs* 96.1	<0.001
	Influence of WASH training	88.0 *vs* 81.7	0.101
Sociodemographic	Sex (female *vs* male)	87.7 *vs* 83.2	0.229
	Grade level	75.0–90.8	0.113

**Notes.**

Percentages represent the proportion of students reporting adequate handwashing (washing hands both before eating and after toilet use) within each category of the explanatory variable. *P*-values are based on Pearson’s chi-square tests.

Variables meeting the inclusion criterion were considered for entry into a multivariable logistic regression model (Table 4). Three factors remained independently associated with adequate handwashing: not relying solely on visible dirt as a cue for handwashing, perceiving the toilet environment as comfortable or safe, and washing hands at least five times per day. WASH education showed a borderline association, whereas knowledge of correct handwashing steps and reporting a broken sink were not significant predictors in the adjusted model.

**Table 4:** Multivariable logistic regression predicting adequate handwashing behaviour (*N* = 368).

Predictor	Adj. OR	95% CI	*p*-value
Routine handwashing (reference: washes only when hands are visibly dirty)	30.3	13.0–71.4	<0.001
Toilet area perceived as comfortable/safe (reference: not comfortable/safe)	6.6	1.8–23.8	0.004
Handwashing ≥5 times per day (reference: <5 times/day)	2.22	1.04–4.74	0.038
Received WASH training (reference: no training)	2.15	0.97–4.76	0.06
Knows correct handwashing steps (reference: does not know)	1.09	0.09–11.0	0.071
Broken sink reported (reference: no broken sink)	0.48	0.12–2.05	0.283

**Notes.**

Adjusted odds ratios were obtained from a multivariable logistic regression model including variables with *p* < 0.20 in bivariate analysis.

## Discussion

This study examined the behavioural determinants of handwashing among students in rural public schools in Georgia using the COM-B framework. Within this framework, the findings indicate that motivational and opportunity-related factors were more strongly associated with adequate handwashing behaviour than capability-related factors. Although knowledge of correct handwashing practices, as a capability-related factor, was high among students, behavioural routines and environmental perceptions played a more important role in predicting consistent handwashing.

The COM-B framework also helped to explain why different types of determinants played unequal roles in predicting behaviour. Capability-related indicators, such as knowledge of handwashing steps, were widely present among students, but this did not necessarily translate into consistent practice. In contrast, motivational factors, particularly habitual routines, and opportunity-related conditions, such as the perceived comfort and safety of hygiene spaces, showed stronger associations with behaviour. This illustrates how the COM-B model helps distinguish behavioural drivers operating at different levels and provides a structured explanation for why knowledge and infrastructure alone may not be sufficient to sustain hygiene practices in school environments.

The strongest predictor was the absence of a “visible dirt” cue as the main trigger for handwashing. Students who did not rely solely on washing their hands when they were visibly dirty had substantially higher odds of adequate handwashing, indicating that stable routines and automaticity may be central to protective hygiene behaviour. This aligns with behavioural research showing that habit strength and environmental cues often outperform knowledge in predicting health-related actions, particularly when individuals are operating under time pressure or facing competing demands typical of school days (Judah et al., 2009; Aunger & Curtis, 2016; Contzen & Mosler, 2015).

The large effect size suggests that interventions shifting handwashing from a reactive behaviour to a routine practice, such as structured class-based handwashing times, consistent prompts, and teacher modelling, could yield meaningful improvements. This finding should be interpreted with caution, as behavioural indicators related to routine handwashing may partially overlap with the operational definition of adequate handwashing used in the study. However, additional model diagnostics did not indicate instability in the regression estimates, suggesting that the large effect size reflects the strength of the behavioural association rather than a statistical artefact.

Perceived comfort and safety of toilet facilities represent an opportunity-related determinant, highlighting the role of the physical and social environment in enabling hygiene behaviour. Even where water is generally available, students may avoid toilets and sinks if the environment feels unpleasant, unsafe, or socially uncomfortable. Prior school-based WASH studies have highlighted the importance of the qualitative experience of sanitation spaces, including privacy, odour, lighting, crowding, cleanliness, and safety, as these factors influence both toilet use and associated hygiene practices (Cronk & Bartram, 2018; Watson et al., 2021). The strong association observed in this study underscores that WASH interventions should include not only functional access, such as water, soap, and working sinks, but also the acceptability and usability of hygiene spaces.

Higher daily handwashing frequency (≥5 times/day) was another independent predictor of adequate handwashing. This likely reflects a broader hygiene orientation, with stronger routines and more consistent exposure to behavioural cues throughout the day. Similar patterns have been reported in other school-based studies, where frequent handwashing correlates with higher compliance at recommended times and may serve as a practical behavioural marker for programme monitoring (Dreibelbis et al., 2016; Figueroa & Kincaid, 2010). Importantly, daily handwashing frequency captures a general hygiene routine and does not necessarily correspond directly to compliance with the two critical moments used to define the outcome. Students may wash their hands frequently during the day but not consistently at the recommended critical times, or vice versa. Therefore, this variable was interpreted as an indicator of habitual hygiene behaviour rather than as a direct measure of the outcome.

In contrast, several capability- and opportunity-related indicators, including knowledge of correct handwashing steps, water availability, soap availability, and general toilet cleanliness, were not independently associated with adequate handwashing after adjustment. This may reflect limited variability in knowledge and perceived importance within the sample (ceiling effect), reducing their predictive value. In addition, once a minimum enabling threshold is met, behavioural drivers such as habit formation, routine cues, and social norms may play a more important role in determining consistent handwashing. This interpretation is consistent with evidence from multiple settings showing that infrastructure and knowledge are necessary but often insufficient without reinforcement through prompts, supervision, and social expectations within the school environment (White et al., 2020; Biran et al., 2014; UNICEF & WHO, 2023).

The borderline association observed for WASH education suggests that school-based hygiene activities may contribute to behaviour, but their impact may depend on how they are delivered. Traditional information-based approaches can raise awareness, yet may not translate into action unless they are combined with behaviourally informed strategies such as environmental prompts, peer engagement, teacher reinforcement, and opportunities to practise routines repeatedly. This is consistent with the intervention literature, which recommends integrating cues and habit-building components into school WASH programmes to support sustained handwashing behaviour (Curtis, Danquah & Aunger, 2009; O’Reilly et al., 2008). The high reported prevalence of adequate handwashing is encouraging for rural schools, where infrastructural and resource constraints are often cited. However, a meaningful minority of students did not meet the adequacy criteria, and the predictors identified suggest practical targets for improvement. Programmes that prioritise routine formation, such as scheduled handwashing times, improve the acceptability and perceived safety of toilet spaces, and incorporate prompts and social reinforcement may strengthen consistent protective hygiene behaviour beyond what knowledge-focused messaging alone can achieve. These findings suggest that motivational drivers and environmental opportunity factors may play a larger role than capability alone in shaping handwashing behaviour among schoolchildren. School schedules, teacher supervision, peer behaviour, and the physical characteristics of sanitation facilities all influence whether handwashing occurs consistently, highlighting the behavioural components that may be most relevant for designing effective school-based hygiene interventions.

The participating schools shared several contextual characteristics typical of rural public schools in the Adjara region, operating within similar public education structures. Basic water access was generally reported by students, although soap availability and infrastructural conditions varied across schools. Some students reported barriers such as broken sinks, unpleasant odours, or discomfort in toilet areas, suggesting variability in the qualitative conditions of sanitation facilities. These environmental differences likely contribute to the strong role of opportunity-related determinants observed in the analysis. However, the relatively small number of participating schools should be considered when interpreting the generalisability of the findings.

### Strengths and limitations

This study applied a theory-driven behavioural framework (COM-B) to examine multiple pathways influencing handwashing behaviour, enabling interpretation beyond infrastructure-only explanations. Nonetheless, several limitations should be considered. The cross-sectional design precludes causal inference, and self-reported handwashing may be subject to social desirability bias, potentially inflating prevalence estimates. Because the questionnaires were interviewer-administered, responses may also have been influenced by interviewer effects. To minimise these risks, interviews were conducted in small groups, students completed their responses individually, and interviewers received standardised training to maintain a neutral tone and avoid suggestive prompts. Nevertheless, some degree of overreporting of recommended hygiene practices cannot be excluded.

The study was conducted in ten rural schools, which may limit the generalisability of the findings to other regions or urban settings. The uneven age distribution of participants means that the findings primarily reflect behavioural patterns among older schoolchildren and adolescents rather than younger students. Future studies including a more balanced age distribution would allow more detailed comparisons across developmental stages.

Some variables, such as water and soap availability, were based on student report rather than direct observation, which may have introduced measurement error. Because participants were recruited from multiple schools, responses may also have been partially correlated within schools. Although sensitivity analyses accounting for school-level clustering did not materially change the findings, the relatively small number of schools limited the use of more complex multilevel modelling. Future studies including a larger number of schools could apply hierarchical models to better account for potential clustering effects.

Despite these limitations, the consistency of the findings with behavioural theory and prior WASH research supports the credibility of the observed associations and highlights actionable targets for school-based interventions in similar contexts.

## Conclusions

This study evaluated the behavioural determinants of adequate handwashing among students in rural public schools in Georgia using the COM-B framework. Although most students reported handwashing at the two key moments, before eating and after toilet use, adequate behaviour was more strongly associated with routine-driven practices, the perceived comfort and safety of toilet spaces, and higher daily handwashing frequency than with knowledge or the basic availability of water and soap. These findings indicate that infrastructure and hygiene knowledge, while necessary, are insufficient on their own to ensure consistent handwashing in school settings.

The study highlights the importance of the behavioural environment within schools, particularly habit formation and the qualitative experience of hygiene spaces, in sustaining protective hygiene practices. However, interpretation is limited by the cross-sectional design and reliance on self-reported behaviour, which may overestimate compliance.

Overall, the findings helped identify whether behavioural gaps are primarily driven by capability, opportunity, or motivational factors, providing useful guidance for the design of school-based WASH interventions.

##  Supplemental Information

10.7717/peerj.21502/supp-1 Supplemental Information 1 STROBE checklist

10.7717/peerj.21502/supp-2 Supplemental Information 2 De-identified dataset and questionnaire (Excel workbook; Adjara, Georgia, 2024) Excel workbook containing de-identified student survey data (participant-level dataset) and the questionnaire items used to generate the dataset. No direct identifiers are included.

## Additional Information and Declarations

### Field Study Permissions

The following information was supplied relating to field study approvals (*i.e*., approving body and any reference numbers): Permission to conduct data collection in the participating schools was obtained verbally from the school principals/administrations of each participating public school in the Adjara region of Georgia; no formal permit number was issued.

### Data Availability

The following information was supplied regarding data availability: The data is available in the Supplemental Files.
